# Supplementary material for: Mechanical compressive forces increase PI3K output signaling in breast and pancreatic cancer cells
Source: Life Sci Alliance. 2025 Jan 2;8(3):e202402854. doi: 10.26508/lsa.202402854 (PMC11707390; doi:10.26508/lsa.202402854)
Supplement: Supplementary file 1 [file LSA-2024-02854_SdataF6_FS2_FS6.pptx]

## Slide 1
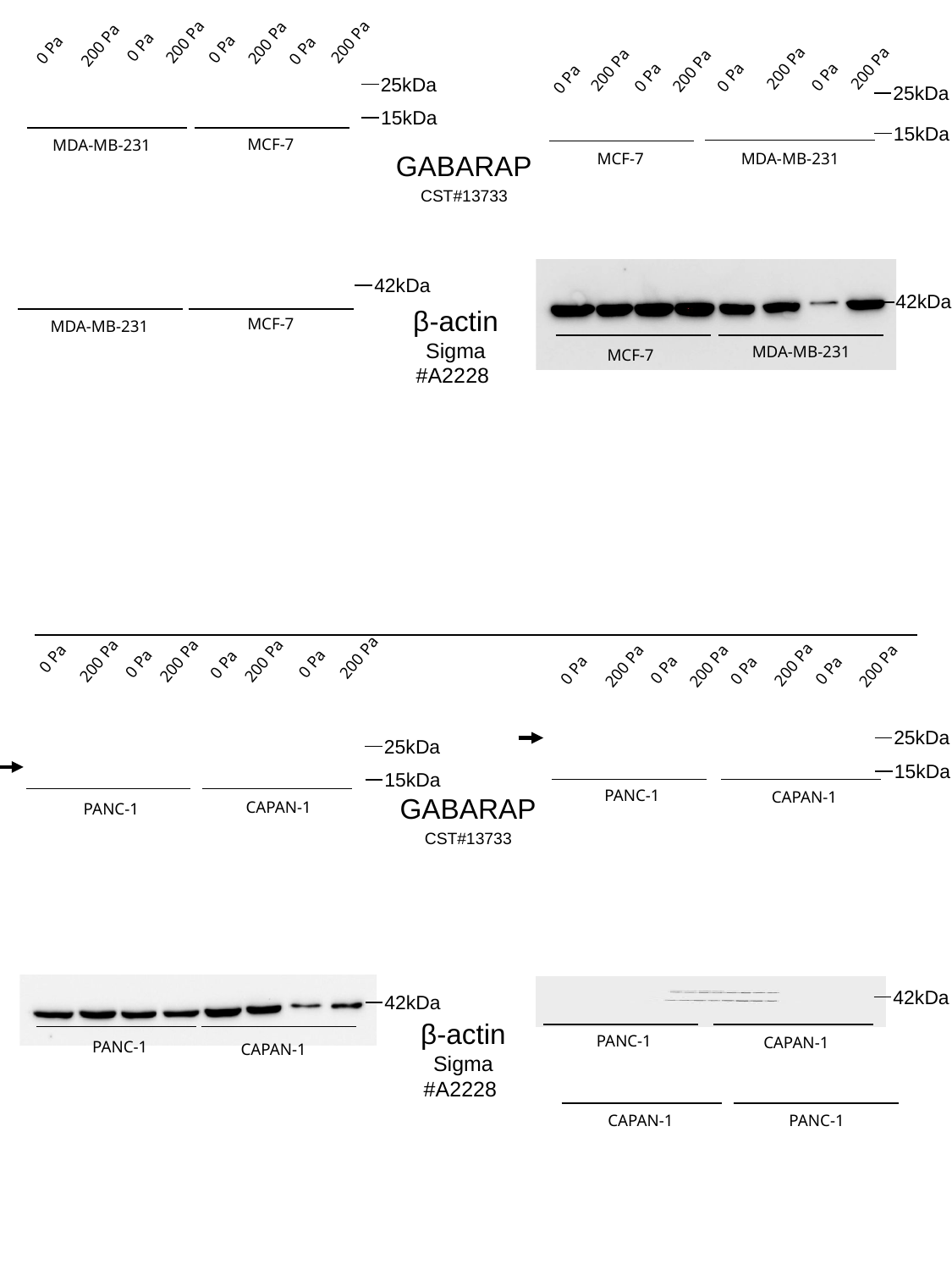

0 Pa
200 Pa
0 Pa
200 Pa
0 Pa
200 Pa
200 Pa
0 Pa
200 Pa
200 Pa
200 Pa
0 Pa
0 Pa
0 Pa
200 Pa
0 Pa
25kDa
25kDa
15kDa
15kDa
MCF-7
MDA-MB-231
GABARAP
CST#13733
MCF-7
MDA-MB-231
42kDa
42kDa
β-actin
Sigma
#A2228
MCF-7
MDA-MB-231
MDA-MB-231
MCF-7
0 Pa
0 Pa
0 Pa
200 Pa
200 Pa
200 Pa
0 Pa
200 Pa
200 Pa
0 Pa
0 Pa
200 Pa
0 Pa
0 Pa
200 Pa
200 Pa
25kDa
25kDa
15kDa
15kDa
PANC-1
CAPAN-1
GABARAP
CST#13733
CAPAN-1
PANC-1
42kDa
42kDa
β-actin
Sigma
#A2228
PANC-1
CAPAN-1
PANC-1
CAPAN-1
CAPAN-1
PANC-1

## Slide 2
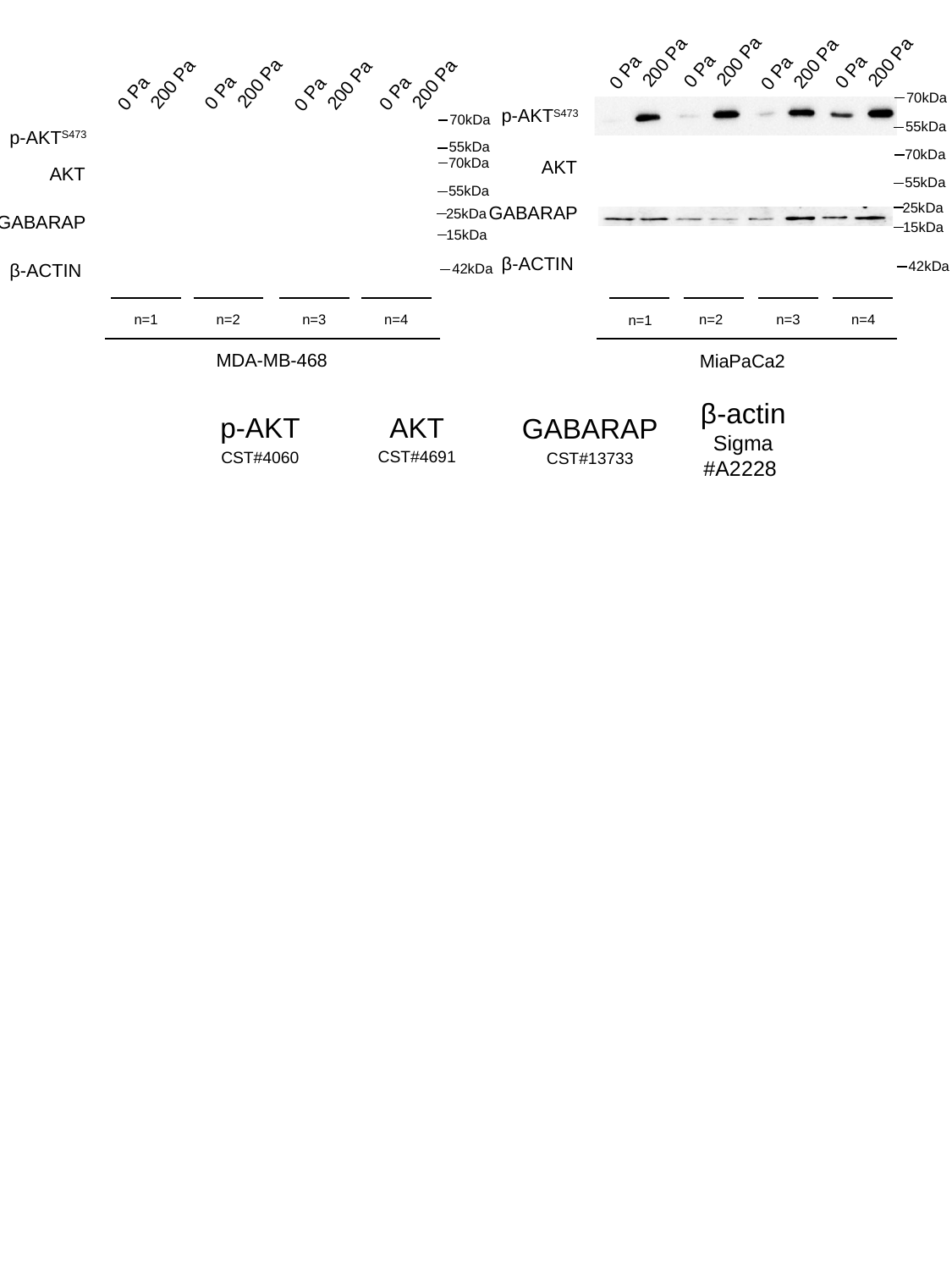

200 Pa
200 Pa
200 Pa
200 Pa
0 Pa
0 Pa
0 Pa
0 Pa
p-AKTS473
AKT
GABARAP
β-ACTIN
n=3
n=4
n=2
n=1
MiaPaCa2
200 Pa
200 Pa
200 Pa
200 Pa
0 Pa
0 Pa
0 Pa
0 Pa
p-AKTS473
AKT
GABARAP
β-ACTIN
n=2
n=4
n=1
n=3
MDA-MB-468
70kDa
70kDa
55kDa
55kDa
70kDa
70kDa
55kDa
55kDa
25kDa
25kDa
15kDa
15kDa
42kDa
42kDa
β-actin
Sigma
#A2228
AKT
CST#4691
p-AKT
CST#4060
GABARAP
CST#13733

## Slide 3
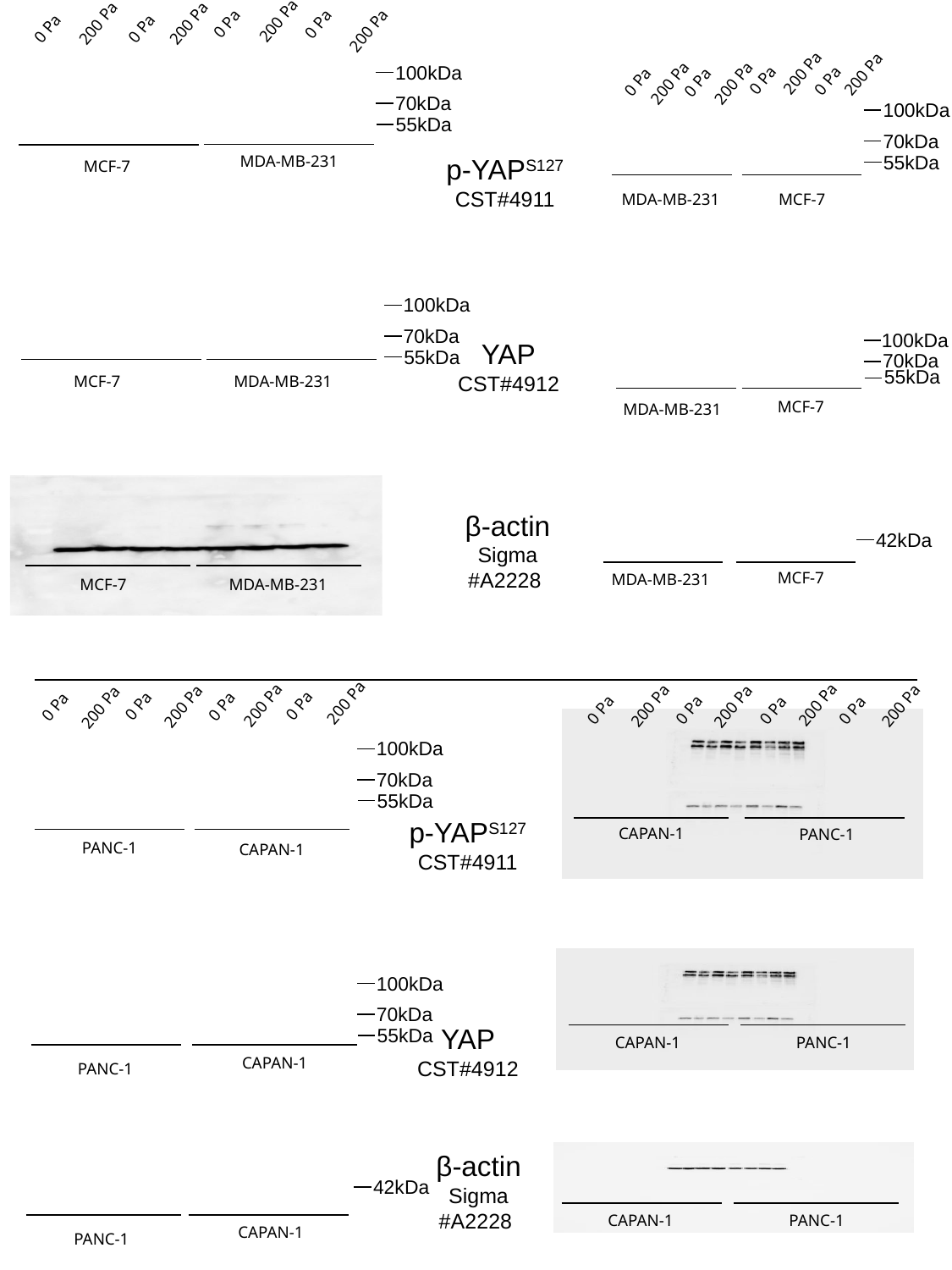

0 Pa
0 Pa
200 Pa
200 Pa
0 Pa
0 Pa
200 Pa
200 Pa
100kDa
200 Pa
0 Pa
0 Pa
200 Pa
0 Pa
0 Pa
200 Pa
200 Pa
70kDa
100kDa
55kDa
70kDa
55kDa
MDA-MB-231
p-YAPS127
CST#4911
MCF-7
MDA-MB-231
MCF-7
100kDa
70kDa
100kDa
YAP
CST#4912
55kDa
70kDa
55kDa
MCF-7
MDA-MB-231
MCF-7
MDA-MB-231
β-actin
Sigma
#A2228
42kDa
MCF-7
MDA-MB-231
MCF-7
MDA-MB-231
0 Pa
0 Pa
0 Pa
0 Pa
200 Pa
200 Pa
200 Pa
0 Pa
0 Pa
200 Pa
0 Pa
0 Pa
200 Pa
200 Pa
200 Pa
200 Pa
100kDa
70kDa
55kDa
p-YAPS127
CST#4911
CAPAN-1
PANC-1
PANC-1
CAPAN-1
100kDa
70kDa
YAP
CST#4912
55kDa
CAPAN-1
PANC-1
CAPAN-1
PANC-1
β-actin
Sigma
#A2228
42kDa
CAPAN-1
PANC-1
CAPAN-1
PANC-1

## Slide 4
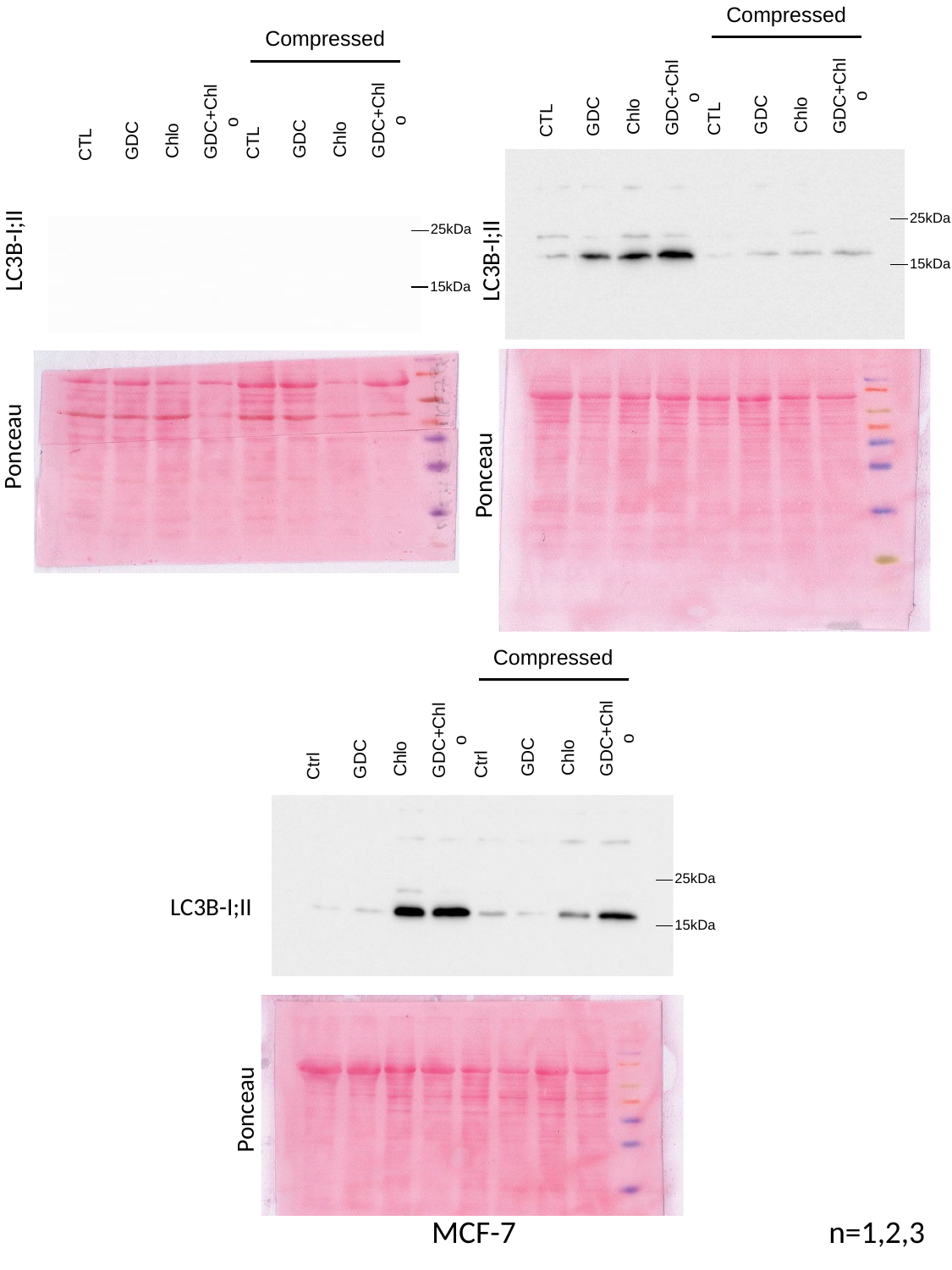

Compressed
GDC+Chlo
GDC+Chlo
GDC
Chlo
GDC
Chlo
CTL
CTL
Compressed
GDC+Chlo
GDC+Chlo
GDC
Chlo
GDC
Chlo
CTL
CTL
25kDa
25kDa
LC3B-I;II
LC3B-I;II
15kDa
15kDa
Ponceau
Ponceau
Compressed
GDC+Chlo
GDC+Chlo
GDC
Chlo
GDC
Chlo
Ctrl
Ctrl
25kDa
LC3B-I;II
15kDa
Ponceau
MCF-7
n=1,2,3

## Slide 5
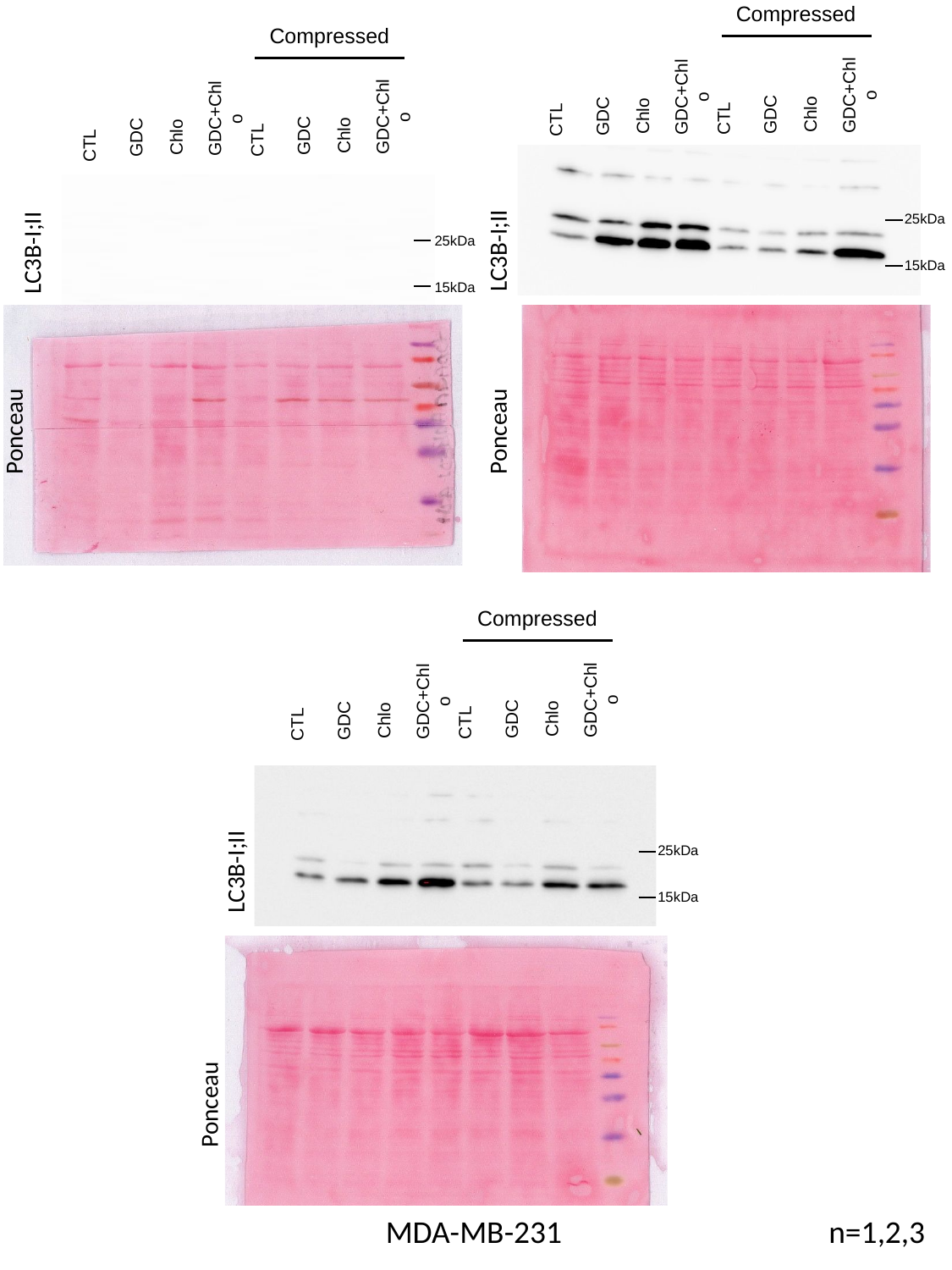

Compressed
GDC+Chlo
GDC+Chlo
GDC
Chlo
GDC
Chlo
CTL
CTL
Compressed
GDC+Chlo
GDC+Chlo
GDC
Chlo
GDC
Chlo
CTL
CTL
25kDa
25kDa
LC3B-I;II
LC3B-I;II
15kDa
15kDa
Ponceau
Ponceau
Compressed
GDC+Chlo
GDC+Chlo
GDC
Chlo
GDC
Chlo
CTL
CTL
25kDa
LC3B-I;II
15kDa
Ponceau
MDA-MB-231
n=1,2,3

## Slide 6
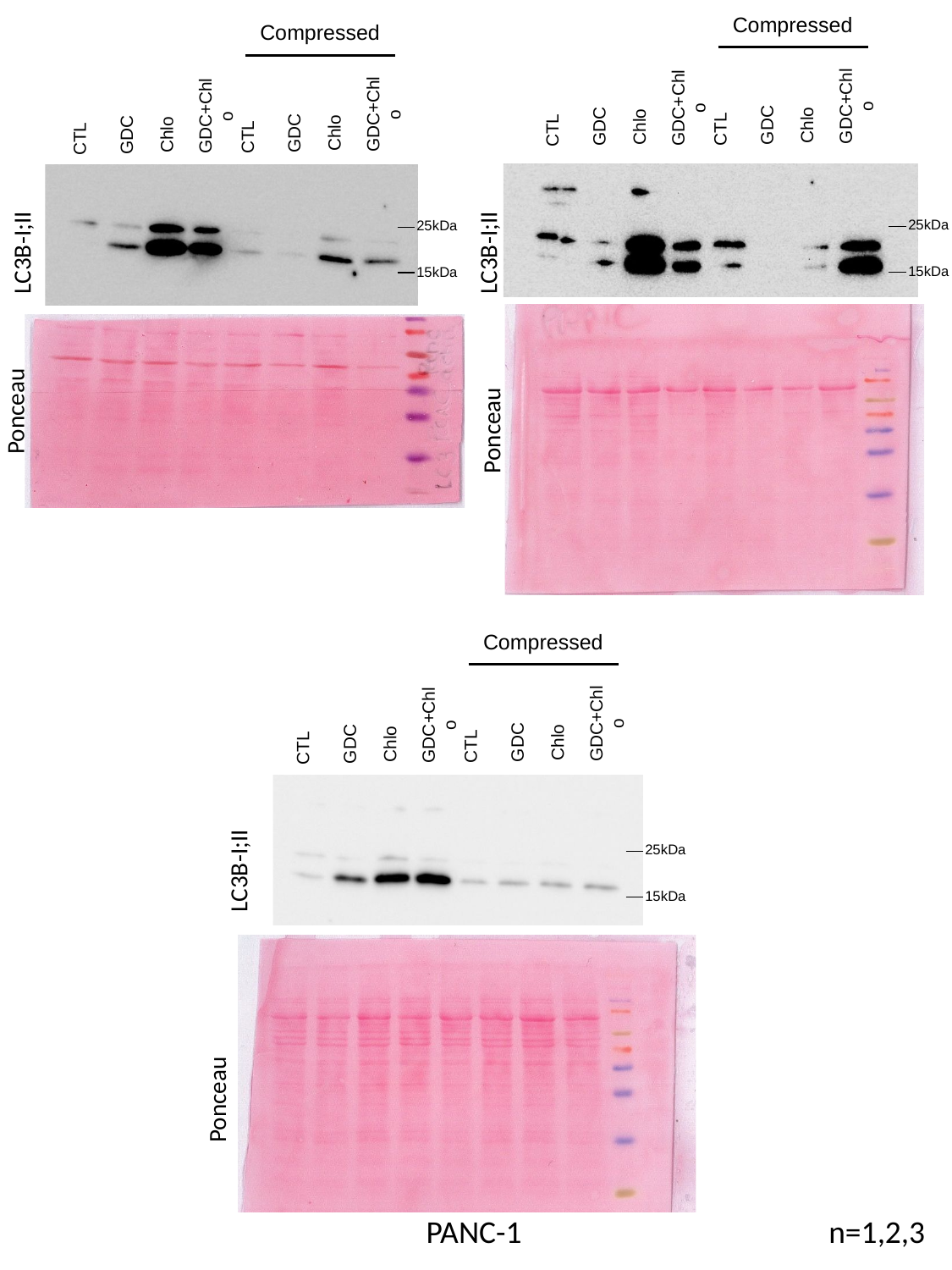

Compressed
GDC+Chlo
GDC+Chlo
GDC
Chlo
GDC
Chlo
CTL
CTL
Compressed
GDC+Chlo
GDC+Chlo
GDC
Chlo
GDC
Chlo
CTL
CTL
25kDa
25kDa
LC3B-I;II
LC3B-I;II
15kDa
15kDa
Ponceau
Ponceau
Compressed
GDC+Chlo
GDC+Chlo
GDC
Chlo
GDC
Chlo
CTL
CTL
LC3B-I;II
25kDa
15kDa
Ponceau
PANC-1
n=1,2,3

## Slide 7
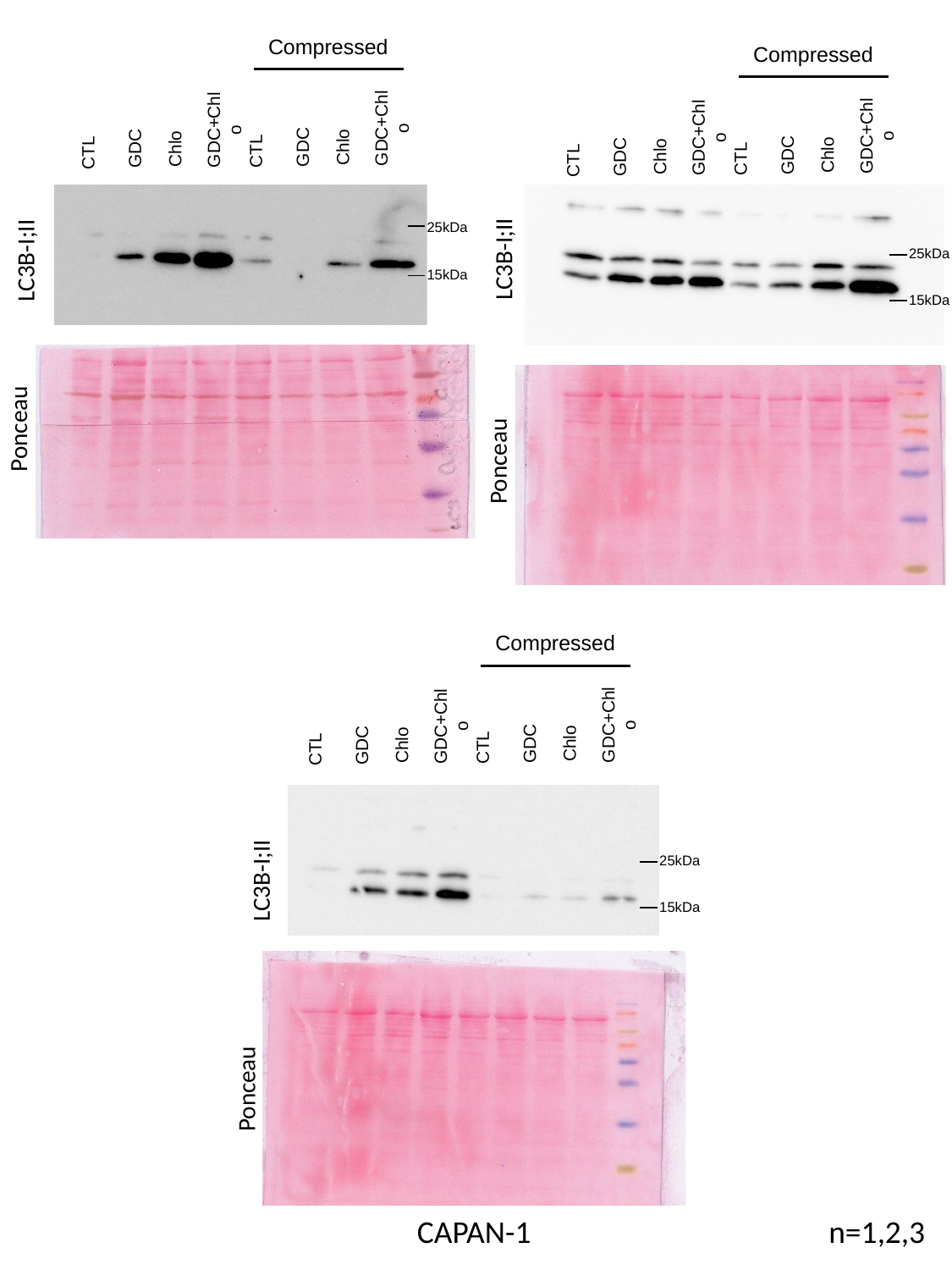

Compressed
GDC+Chlo
GDC+Chlo
GDC
Chlo
GDC
Chlo
CTL
CTL
Compressed
GDC+Chlo
GDC+Chlo
GDC
Chlo
GDC
Chlo
CTL
CTL
25kDa
LC3B-I;II
25kDa
LC3B-I;II
15kDa
15kDa
Ponceau
Ponceau
Compressed
GDC+Chlo
GDC+Chlo
GDC
Chlo
GDC
Chlo
CTL
CTL
LC3B-I;II
25kDa
15kDa
Ponceau
CAPAN-1
n=1,2,3
