# Supplementary material for: Mechanical compressive forces increase PI3K output signaling in breast and pancreatic cancer cells
Source: Life Sci Alliance. 2025 Jan 2;8(3):e202402854. doi: 10.26508/lsa.202402854 (PMC11707390; doi:10.26508/lsa.202402854)
Supplement: Supplementary file 2 [file LSA-2024-02854_TableS1.docx]

# Supporting Tables

## Supporting Table 1. List of primers

| **APPLICATION** | **GENE** | **SEQUENCE** |
| --- | --- | --- |
| RT-qPCR | Homo Sapiens |  |
| **Housekeeping gene** | *ACTB* | Forward: 5’-CTCCATCATGAAGTGTGACGTG-3' |
|  |  | Reverse: 5’-GGAGTACTTGCGCTCAGG-3' |
| **PI3K pathway** | *PIK3CA* | Forward: 5'-GTATCCCGAGAAGCAGGATTTAG -3' |
|  |  | Reverse: 5'-CAGAGAGAGGATCTCGTGTAGAA-3‘ |
|  | *PIK3CB* | Forward: 5'-ATGGGTGAGCCTCTTCTTTATG -3' |
|  |  | Reverse: 5'-CCTATTCCTGAGGTTGGTTTGT-3' |
|  | *PIK3CD* | Forward: 5'-CCCACAGGTGATCCTAACATATC-3' |
|  |  | Reverse: 5'-ACTTCTGGCTCTGTTGAGTTT-3' |
|  | *PIK3CG* | Forward: 5'- GATTCTTCTTCCTTGCCCTTTG-3' |
|  |  | Reverse: 5'-CAGGTGGGTAGAGTGTGATTT-3' |
| **Autophagy pathway** | *GABARAPL1* | Forward: 5'-CCAGTACAAGGAGGACCATCC-3' |
|  |  | Reverse: 5'-GAATAAGGCGTCCTCAGGTCTC-3' |
| **YAP/TAZ pathway** | *TEAD1* | Forward: 5’-CAGGAGGAGACTCTCCCTG-3’ |
|  |  | Reverse: 5’-CCTCCTGAAAGCTTTGCTCG-3’ |
|  | *c-JUN* | Forward: 5'-GGTCGGCAGTATAGTCCGAAC-3' |
|  |  | Reverse: 5'-CTTTCCGCCGCTGTCAAC-3' |
|  | *CCN2* | Forward: 5’-CCTATTCTGTCACTTCGGCTC-3’ |
|  |  | Reverse: 5’-CAGACGAACGTCCATGCTG-3’ |
|  | *CCN1* | Forward: 5'-GCTCTGAAGGGGATCTGC-3' |
|  |  | Reverse: 5'-GTAACTTTGACCAGCCGAGG-3' |
| **ShRNA** | Homo Sapiens |  |
|  | *Sh-RNA-Scramble* | Forward:  5′CGCGTCCCCTTCTAGAGATAGTCTGTACGTTTCAAGAGAACGTACAGACTATCTCTAGAATTTTTGGAAAT-3’ |
|  |  | Reverse:  5′-CGATTTCCAAAAATTCTAGAGATAGTCTGTACGTTCTCTTGAAACGTAC AGACTATCTCTAGAAGGGGA |
|  | *Sh-RNA-p110α1* | Forward:  5’-CGCGTCCCCGCGAAATTCTCACACTATTATTTCAAGAGAATAATAGTGT GAGAATTTCGCTTTTTGGAAAT-3’ |
|  |  | Reverse:  5'- CGATTTCCAAAAAGCGAAATTCTCACACTATTATTCTCTTGAAATAAT AGTGTGA-GAATTTCGCGGGGA |
|  | *Sh-RNA-p110α2* | Forward:  5’-CGCGTCCCCGCACAATCCATGAACACATTTTCAAGGAAATGCTGTTCA TGGATTGTGCTTTTTGGAAAT |
|  |  | Reverse:  5'- CGATTTCCAAAAAGCACAATCCATGAACACATTTCTCTGAAAATGCTG TTCATGGATTGTGCGGGGA-3’ |
