# Supplementary material for: Mechanical compressive forces increase PI3K output signaling in breast and pancreatic cancer cells
Source: Life Sci Alliance. 2025 Jan 2;8(3):e202402854. doi: 10.26508/lsa.202402854 (PMC11707390; doi:10.26508/lsa.202402854)
Supplement: Supplementary file 3 [file LSA-2024-02854_TableS2.docx]

## Supporting Table 2. Primary antibodies

| **PRIMARY ANTIBODY** | **SPECIES** | **SOURCE** | **REFERENCE NUMBER** | **DILUTION IF/WB** |
| --- | --- | --- | --- | --- |
| β-ACTIN | Mouse | Sigma Aldrich | #A2228 | 1/10000 |
| AKT | Rabbit | Cell Signaling | #4691 | 1/1000 |
| GABARAP | Rabbit | Cell Signaling | #13733 | 1/1000 |
| LC3B | Rabbit | Cell Signaling | #2775 | 1/1000 |
| p110α | Rabbit | Cell Signaling | #4249 | 1/500 |
| p110β | Rabbit | Santa Cruz Biotechnology | #602 | 1/500 |
| p62/SQSTM1 | Rabbit | Cell Signaling | #7695 | IF: 1/500 |
| p-AKT(Ser473) | Rabbit | Cell Signaling | #4060 | 1/2000 |
| p-YAP(Ser127) | Rabbit | Cell Signaling | #4911 | 1/1000 |
| YAP | Rabbit | Cell Signaling | #4912 | 1/1000 |
