# Supplementary material for: Mechanical compressive forces increase PI3K output signaling in breast and pancreatic cancer cells
Source: Life Sci Alliance. 2025 Jan 2;8(3):e202402854. doi: 10.26508/lsa.202402854 (PMC11707390; doi:10.26508/lsa.202402854)
Supplement: Supplementary file 4 [file LSA-2024-02854_TableS3.docx]

## Supporting Table 3. Secondary antibodies

| **SECONDARY ANTIBODY** | **SPECIES** | **SOURCE** | **REFERENCE NUMBER** | **DILUTION WB** |
| --- | --- | --- | --- | --- |
| Anti-rabbit IgG-Horse Radish Peroxidase | Goat | Invitrogen | #31460 | 1/5000 |
| Anti-mouse IgG-Horse Radish Peroxidase | Goat | Invitrogen | #31430 | 1/10000 |
| Anti-rabbit Alexa Fluor 488 | Goat | Abcam | #ab150077 | 1/200 |
